# Resveratrol suppresses the growth and metastatic potential of cervical cancer by inhibiting STAT3^Tyr705^ phosphorylation

**DOI:** 10.1002/cam4.3510

**Published:** 2020-10-10

**Authors:** Xiaodong Sun, Qianqian Xu, Lian Zeng, Lixia Xie, Qiang Zhao, Hongxia Xu, Xuanbin Wang, Nan Jiang, Pan Fu, Ming Sang

**Affiliations:** ^1^ Hubei Institute of Parkinson's Disease at Xiangyang No. 1 People’s Hospital Hubei Key Laboratory of Wudang Local Chinese Medicine Research Hubei University of Medicine Shiyan People’s Republic of China; ^2^ Hubei Province Hospital of Traditional Chinese Medicine Hubei Province Academy of Traditional Chinese Medicine Wuhan People’s Republic of China

**Keywords:** cervical cancer, epithelial‐mesenchymal transition, extracellular matrix, resveratrol, STAT3 phosphorylation

## Abstract

Aberrant signal transducer and activator of transcription 3 (STAT3) signaling promotes the initiation and progression of cancer in humans by either inhibiting apoptosis or inducing cell proliferation, angiogenesis, invasion, and metastasis. The role of resveratrol（RES）in inhibiting the STAT3 signaling pathway *in vivo*, particularly in cervical cancer is still unknown. This study aims to investigate the role of STAT3 and its phosphorylation in RES‐mediated suppression of cervical cancer. The effects of RES on cervical cancer were determined by examining tumor tissues, their histological changes, and the volume and weight of tumor tissues grown from HeLa cells injected in female athymic BALB/C nude mice. The structure and target interaction of RES were virtually screened using the molecular docking program Autodock Vina. The status of phosphorylated STAT3, protein levels of epithelial‐mesenchymal transition molecular markers and extracellular matrix degradation enzymes were determined through Western blot. We demonstrated that RES could suppress the proliferation and metastatic potential of cervical cancer cells by inactivating phosphorylation of STAT3 at Tyr705 but not Ser727. This effect was intensified by inhibition of the STAT3 signal pathway.

## Graphical abstract

Resveratrol inhibits growth and metastatic potential of cervical cancer *in vivo* and *in vitro*. Resveratrol inhibits phosphorylation of STAT3 at Tyr705 but not Ser727. Resveratrol Suppress growth and Epithelial Mesenchymal Transition of cervical cancer through inhibiting STAT3 Tyr705 phosphorylation.

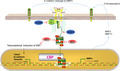

**Abbreviations**
CC: cervical cancer
CCK‐8: cell counting kit‐8 assay
DAB: diaminobenzidine
ECL: electrochemiluminescence
ECM: extracellular matrix
EMT: epithelial‐mesenchymal transition
FBS: fetal bovine serum
GAPDH: glyceraldehyde 3‐phosphate dehydrogenase
H&E: hematoxylin‐eosin staining
HPV: human papilloma virus
IHC: immunohistochemistry
IL‐6: interleukin‐6
MMP‐13: matrix metalloproteinase 13
MMP‐3: matrix metalloproteinase 3
qRT‐PCR: quantitative reverse transcription‐polymerase chain reaction
RES: resveratrol
RIPA: radioimmunoprecipitation assay
SDS: Sodium dodecyl sulfate
STATs: signal transducers and activators of transcription
STR: short tandem repeat
TBS: triethanolamine‐buffered saline solution
TBST: TBS + Tween

## 1 INTRODUCTION

Cervical cancer is one of the most common malignant tumors affecting women worldwide. The survival outcome of patients with cervical cancer receiving adjuvant chemotherapy and/or adjuvant radiotherapy (chemoradiotherapy) after radical hysterectomy are similar, and the risk of distant recurrence is reduced.^1^ Neoadjuvant chemotherapy followed by radical surgery also has a favorable prognosis for locally advanced cervical cancer.^2^, ^3^ The main chemotherapy regimens for this disease include platinum‐based drugs and taxanes. However, new drugs need to be developed due to drug resistance and adverse reactions observed with the current treatment.^4^, ^5^

Resveratrol (RES) is a natural stilbene and nonflavonoid polyphenol that possesses antioxidant, anti‐inflammatory, cardioprotective, and anticancer properties. It also has beneficial effects on breast, cervical, blood, kidney, liver, bladder, thyroid, prostate, brain, lung, gastric, colon, head and neck, and bone cancers.^6^, ^7^, ^8^ In addition, RES can reverse multidrug resistance in cancer cells and sensitize them to standard chemotherapeutic agents.^6^, ^7^, ^8^ RES induces autophagy and apoptosis of cervical cancer cells^9^, ^10^ and suppresses the migration and invasion of human cervical cancer cells.^11^ RES also significantly inhibits the occurrence and development of cervical cancer by regulating phospholipid scramblase 1^12^, ^13^ and exhibits antitumor activity on human papilloma virus (HPV) E6‐positive cervical cancer.^14^, ^15^ Therefore, RES is a potential chemotherapeutic drug for cervical cancer.

Signal transducers and activators of transcription (STATs) constitute a family of cytoplasmic transcription factors that mediate intracellular signaling from cell surface receptors to the nucleus, transactivate genes encoding apoptosis inhibitors and cell cycle regulators, and induce angiogenesis. STAT3 is activated in a wide variety of human tumors, including breast, lung, gastric, hepatocellular, colorectal, and prostate cancers. Aberrant STAT3 signaling promotes the initiation and progression of human cancers by either inhibiting apoptosis or inducing cell proliferation, angiogenesis, invasion, and metastasis. STAT3 activity suppression induces the apoptosis of tumor cells.^13^, ^16^, ^17^ RES inhibits the interleukin‐6 (IL‐6)‐induced transcriptional activity of STAT3 in human prostate cancer LNCaP‐FGC cells^18^ and STAT3 axis in primary glioblastoma tumor‐initiating cells.^19^ STAT3 signaling inhibition plays a critical role in the RES‐induced suppression of several cancer types, including ovarian cancer,^20^, ^21^ pancreatic cancer,^22^ head and neck tumor,^23^ osteosarcoma,^24^ colorectal cancer,^25^ and colon cancer.^26^ In these cancer types, RES inhibits STAT3 ^Tyr705^ ^22^, ^23^, ^24^, ^25^, ^26^ and STAT3^S727^ ^23^, ^25^ phosphorylation. Zhang et al.^27^ showed that STAT3 signaling is critical for cervical cancer cells and the major target for RES because selective STAT3 inhibition, rather than Wnt or Notch activation causes SiHa and HeLa cells to undergo apoptosis. However, how RES exerts antitumor effects on cervical cancer cells by inhibiting STAT3 phosphorylation *in vivo* remains unknown.

In this study, the STAT3 phosphorylation status in cervical cancer cells and a mouse xenograft tumor model was investigated after RES treatment. Our results suggest that RES inhibited the growth and metastatic potential of cervical cancer by suppressing STAT3 ^Tyr705^ phosphorylation. Therefore, RES could be used as a chemotherapeutic drug for cervical cancer.

## 2 METHODS

### 2.1 Cell culture

HPV18‐positive HeLa cells^28^ and HPV16‐positive SiHa cells,^29^, ^30^, ^31^ that are human cervical carcinoma cell lines, were purchased from Hunan Fenghui Biological Technology Co., Ltd. (Hunan, China). These cells were certified via short tandem repeat (STR) analysis. HeLa and SiHa cells were cultured in Dulbecco's Modified Eagle Medium (DMEM, Gibco, 11965‐092) containing 10% of fetal bovine serum (FBS, Capricorn, FBS‐HI‐11A), 100 IU/ml of penicillin G sodium, and 100 mg/ml of streptomycin sulfate at 37 °C in an incubator with 5% CO_2_/95% air humidified atmosphere. Cells in the exponential growth phase were used in these experiments.

### 2.2 Animal model and in vivo antitumor efficacy of res

**Figure 1:**
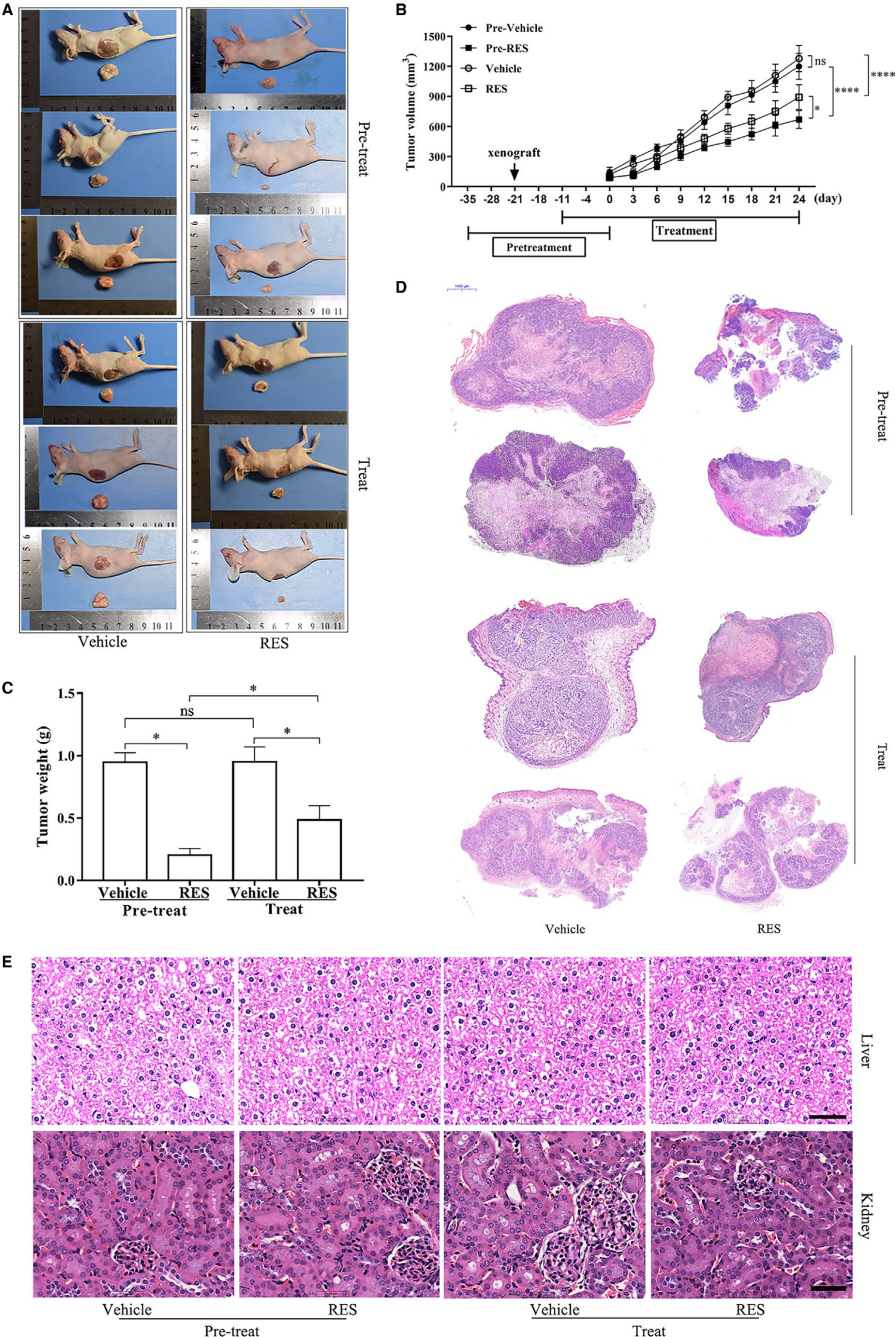
RES inhibits the growth of cervical cancer in a mouse model. HeLa cells were injected into female athymic BALB/C nude mice, who underwent pretreatment and treatment regimen (n = 6/group) with RES. The size (A), volume (B), and weight (C) of the tumor tissues grown from HeLa cells were examined and measured. Tumor tissues were then histologically examined with hematoxylin and eosin staining (D). *, *p* < 0.05; ****, *p* < 0.0001. (E) The main organs (liver and kidney) of tumor‐bearing nude mice after treatment were histologically examined with hematoxylin and eosin staining, scale bar =25 μm

Twenty‐four female athymic BALB/C nude mice weighing 14‐20 g (4‐6 weeks) were purchased from Hunan SJA Laboratory Animal Co., Ltd. (Changsha, China). The mice were housed at 20°C‐22°C with 50%‐60% relative humidity and fed with standard laboratory chow and tap water *ad libitum*. About 24 mice were randomly divided into two regimens and given the pretreatment and treatment. In the pretreatment regimen, 12 mice were randomly divided into control group administered vehicle (normal saline containing 0.1% ethanol, 3 times/week, intragastric administration), and pretreatment group administered RES (30 mg/kg, 3 times/week, intragastric administration). The 12 mice were pretreated for 2 weeks. After 2 weeks, 24 mice were subcutaneously injected in the right flank with 200 μl of HeLa cell suspensions containing 5 × 10^6^ cells in sterile saline. In the pretreatment regimen, 12 mice were treated with vehicle and RES for 3 weeks sequentially, and were raised to the end of the experimental process. In the treatment regimen, 12 mice were divided into control group and treatment group according to tumor volume after 10 days of HeLa cell injection, the drug dosage, and administration method were consistent with the pretreatment regimen, and the treatment lasted for 5 weeks. The key time nodes are explained in Figure 1B. Food, water intake, and behavioral changes were monitored daily, the body weight and tumor volumes were recorded every 3 days throughout the test period. Tumor volumes were calculated with the tumor length and width, which were measured using a caliper: tumor volume = (length) × (width)^2^ × 0.5. At the end of the treatment, all the mice were sacrificed by cervical dislocation. Tumors were isolated, weighed, and aliquoted for Western blot analysis, hematoxylin‐eosin (H&E) staining, and immunohistochemical (IHC) staining assay. This study was approved by the ethical committee for animal experimentation of Xiangyang No. 1 People's Hospital (NO. 2017DW006).

### 2.3 Determination of cell proliferation using the CCK‐8 assay

HeLa cells were seeded in 96‐well plates at a density of 10,000 cells/well, incubated at 37°C for 30 min, treated with RES (Sigma–Aldrich, 1602105‐100MG, USA) or the control vehicle, and cultured at 37°C for 24, 48, and 72 h. Afterward, they were washed once with 125 μl of phosphate‐buffered saline (PBS)/well and assayed using a cell counting kit‐8 (CCK‐8) kit (Dojindo Laboratories, Kumamoto, Japan) in accordance with the manufacturer's instructions. The 96‐well plates were read at 450 nm on a plate reader (SpectraMax®iD3, Molecular Devices, USA). Cell viability was denoted by the percentage of cell loss, which was calculated using the following formula: (Drug_A450_/Control_A450_) × 100, where A450 denotes the absorbance at a wavelength of 450 nm.

### 2.4 Colony formation assay

A suspension of individualized HeLa cells was prepared from the cultured cells through trypsin digestion and pipetting. The cell suspension was diluted with DMEM containing 10% of FBS and the desired concentrations of RES or vehicle control and then aliquoted to six‐well plates at a density of 100 cells/well. The cells were cultured for 14 days, washed with cold PBS twice, and fixed with 3.7% of formaldehyde. Then, the cell colonies were stained with crystal violet (Sinopharm Chemical Reagent Co., Shanghai, China), and the number of colonies in each well was counted.

### 2.5 Wound healing assay

The effects of RES on the migration of HeLa and SiHa cells were examined using wound healing assay. HeLa cells were seeded in a 6‐well plate at a density of 5 × 10^5^ cells/well. A scratch was made across the cell monolayer on the bottom of the plates with a 200 μl sterile pipette tip and washed with PBS on reaching 80% confluence. The cultures were then treated with RES at the indicated concentrations or untreated (blank control) and incubated for 0, 24, 48, and 72 h. The images of cultures were taken with an inverted microscope (Olympus Optical Co., Ltd., IX73P2F, Japan). The scratches across the cell culture were measured on the basis of the images by using Olympus cellSens (Olympus, Version 1.5, Japan). The experiment was repeated thrice.

### 2.6 Invasion assay

The effects of RES on the invasion of HeLa and SiHa cells were examined using a Transwell assay. Boyden chambers containing 24‐well Transwell plates (Corning Inc., USA) with 8 mm pore size were used. HeLa cells were seeded at a density of 1 × 10^5^ cells/ml in the upper chambers coated with Matrigel and treated with 0, 10, 20, and 40 μM RES, dissolved in a medium. DMEM containing 10% of FBS was added to the bottom chamber. After the cells were cultured for 24 h, the filters in the upper chambers were collected. Cells on the upper side of the filter membrane were wiped with a cotton swab, and the cells invading the lower side of the filter membrane were fixed in 4% of paraformaldehyde to the slides, and stained with 0.1% of crystal violet for 10 min (Sinopharm Chemical Reagent Co., Shanghai, China). The cells in the slides were examined and counted in five randomly selected microscopic fields (×400) by using an inverted microscope (IX73P2F, Olympus Optical Co., Ltd., Japan). The number of cells was compared in each treatment group. All the experiments were performed in duplicate and repeated thrice.

### 2.7 Western blot analysis

HeLa and SiHa cells were collected and homogenized in a radioimmunoprecipitation assay (RIPA) lysis buffer (Beyotime Biotechnology, P0013B) after they were treated with the corresponding drugs. Tissue samples were also homogenized in a RIPA. The homogenized samples were then centrifuged at 12,000 × *g* and 4°C for 15 min, and the supernatants were collected. An aliquot of the supernatant was used to determine the protein concentration by using a Bio‐Rad DC protein assay kit (Bio‐Rad Laboratories, Hercules, CA). All the samples containing 30 μg of protein/sample were aliquoted, mixed with 5× loading buffer and loaded for electrophoresis in 10% of SDS‐polyacrylamide gel. The resolved proteins in the gel were transferred onto polyvinylidene difluoride membranes after electrophoresis. The membranes were blocked with 5% of nonfat milk TBST buffer (20 mM Tris pH 7.4, 150 mM NaCl, and 0.1% Tween‐20) at room temperature for 2 h, probed with primary antibodies overnight at 4 °C, washed with TBST buffer thrice, incubated with the corresponding horseradish peroxidase‐conjugated secondary antibody (1:10,000 dilution, Santa Cruz Biotechnology, Santa Cruz, CA, USA) at room temperature for 1 h, and developed using an ECL substrate (Thermo Fisher Scientific, Waltham, MA, USA). The relative density of the blots was quantified using Lab Works (UVP, Upland, CA, USA). β‐actin or glyceraldehyde 3‐phosphate dehydrogenase (GAPDH) was used as the loading control. The relative expression of target proteins was normalized with the loading control. The sources and dilution of primary antibodies were as follows: E‐cadherin (4A2) mouse mAb (#14472), N‐cadherin (D4R1H) rabbit mAb (#13116), and vimentin (D21H3) XP® rabbit mAb (#5741; Cell Signaling Technology, Inc.); GAPDH antibody (Absin, abs132004), β‐actin antibody (Absin, abs132001), MMP‐3 antibody (Absin, abs135854), MMP‐13 antibody(Absin, abs110501), rabbit antihuman phospho‐Stat3‐Y705 polyclonal antibody (Absin, abs118973), phospho‐Stat3(Absin, Ser727) antibody (Absin, abs130919), and STAT3 antibody (Absin, abs131812; Absin Bioscience Co., Ltd., Shanghai, China). The primary antibodies were diluted at a 1:1000 ratio. A ChemiDoc™ MP imaging system with Image Lab™ (version 5.1, Bio‐Rad Laboratories, Inc., USA) was used as the image acquisition tool and image processing software package.

### 2.8 Molecular docking

Structure‐based virtual screening was conducted in the molecular docking program Autodock Vina version 1.1.2.^32^ The 3D schematic of protein‐ligand macromolecules was generated with PyMol version 2.3.^33^ The 2D schematic of the interaction between ligands and other amino acid residues was drawn with LigPlus version 2.1.^34^ The structure file of the STAT3 protein (PDB ID:6QHD) was extracted from the Research Collaboratory for Structural Bioinformatics Protein Data Bank (http://www.rcsb.org/).^35^, ^36^ The protein is an X‐ray crystal homodimer structure bound to the DNA, at 2.85 Å resolution.^37^ All heteroatom, including double‐stranded DNA and crystallographic water were removed, and chain A was kept as a STAT3 monomer. The native pTyr peptide in the 6QHD crystal structure of the monomer ligand was removed to examine whether RES competitively bound to the pTyr705 peptide pocket. The *trans*‐RES molecular structure (PubChem CID: 445154) was obtained from the PubChem database.^38^ The Autodock Vina tutorial was followed to convert the ligand and receptor pdb to a pdbqt file by using AutoDock Tools.^39^ The grid sizes in XYZ were set at 96, 66, and 118, and were large enough to contain all the potential pockets in the STAT3 monomer. The pocket with the lowest score that predicted the highest binding affinity was chosen as the RES binding site in STAT3.

### 2.9 H&E staining and IHC assay

Tumor tissues were harvested, fixed with 4% of formaldehyde, embedded with paraffin, and cut into 4 µm thick sections. For histological examination of tumor tissues, the paraffin‐embedded sections were subjected to H&E staining and examined under an inverted microscope (Olympus IX73). For the IHC assay of the expression levels of p‐STAT3 ^Tyr705^, E‐cadherin, N‐cadherin, and vimentin, the paraffin‐embedded sections were incubated with antihuman STAT3, p‐STAT3 ^Tyr705^, E‐cadherin, N‐cadherin, and vimentin primary antibodies, and a biotinylated goat anti‐rabbit antibody was used as a secondary antibody. Then, the slides were washed with PBS and incubated with diaminobenzidine chromogen for 3‐5 min to yield a dark brown specimen. The sections were counterstained with hematoxylin for microscopic observation (IX73P2F, Olympus Optical Co., Ltd., Japan). Cells with moderate and strong brownish cytoplasmic staining were considered positive, whereas cells with unstained or weakly stained cytoplasm were considered negative. The expression levels of STAT3, p‐STAT3 ^Tyr705^, E‐cadherin, N‐cadherin, and vimentin were determined by calculating the ratio of the number of positively stained cells to the total number of cells in five randomly selected microscopic fields at 400× magnification, using Image J software (National Institutes of Health).

### 2.10 Statistical analysis

Data were analyzed using GraphPad Prism 7.0 (GraphPad Software, San Diego, CA) software. Results were expressed as mean ±standard deviation for at least three independent experiments. Statistical comparisons between groups were performed using Student's *t* test or one‐way ANOVA, followed by a post hoc Student‐Newman‐Keuls test. Data with *p* < 0.05 were considered statistically significant.

## 3 RESULTS

### 3.1 Cervical tumor growth inhibition in mice using RES

The tumor tissues, their histological changes, and the volume and weight of tumor tissues grown from HeLa cells injected in female athymic BALB/C nude mice were examined after undergoing pretreatment and treatment with RES, to determine the effects of RES on cervical tumor growth *in vivo*. The results showed that the size (Figure 1A), volume (Figure 1B), and weight (Figure 1C) of tumor tissues significantly decreased in the RES pretreatment and treatment groups compared to those in their respective control groups. The RES pretreatment and treatment regimen damaged the tumor mass, as revealed by H&E staining (Figure 1D). In addition, H&E staining of mice organs after treatment did not show any significant signs of toxicity or inflammatory lesions (Figure 1E). The magnitude of the changes in the volume (Figure 1B), weight (Figure 1C), and histological characteristics of tumor tissues (Figure 1D,E) was higher in the RES pretreatment group than in the RES treatment group. These results suggested that RES inhibited cervical tumor growth and prevented the occurrence of cervical cancer in the mouse model.

### 3.2 RES inhibits the proliferation of cervical cancer cells

HeLa cells were treated with RES and their cell proliferation was determined through CCK‐8 and colony formation assays to examine the effect of RES on the proliferation of cervical cancer cells. The proliferation of HeLa cells was inhibited by RES in a dose‐dependent manner (Figure S1A). RES had IC_50_ of 291.3, 50.09, and 8.73 μΜ in HeLa cells for 24, 48, and 72 h, respectively (Figure S1B). The number of HeLa cell colonies decreased with RES treatment in a dose‐dependent manner (Figure S1C,D). These results confirmed that RES inhibited the proliferation of cervical cancer cells in a dose‐ and time‐dependent manner.

### 3.3 RES suppresses the migration and invasion of cervical cancer cells

**Figure 2:**
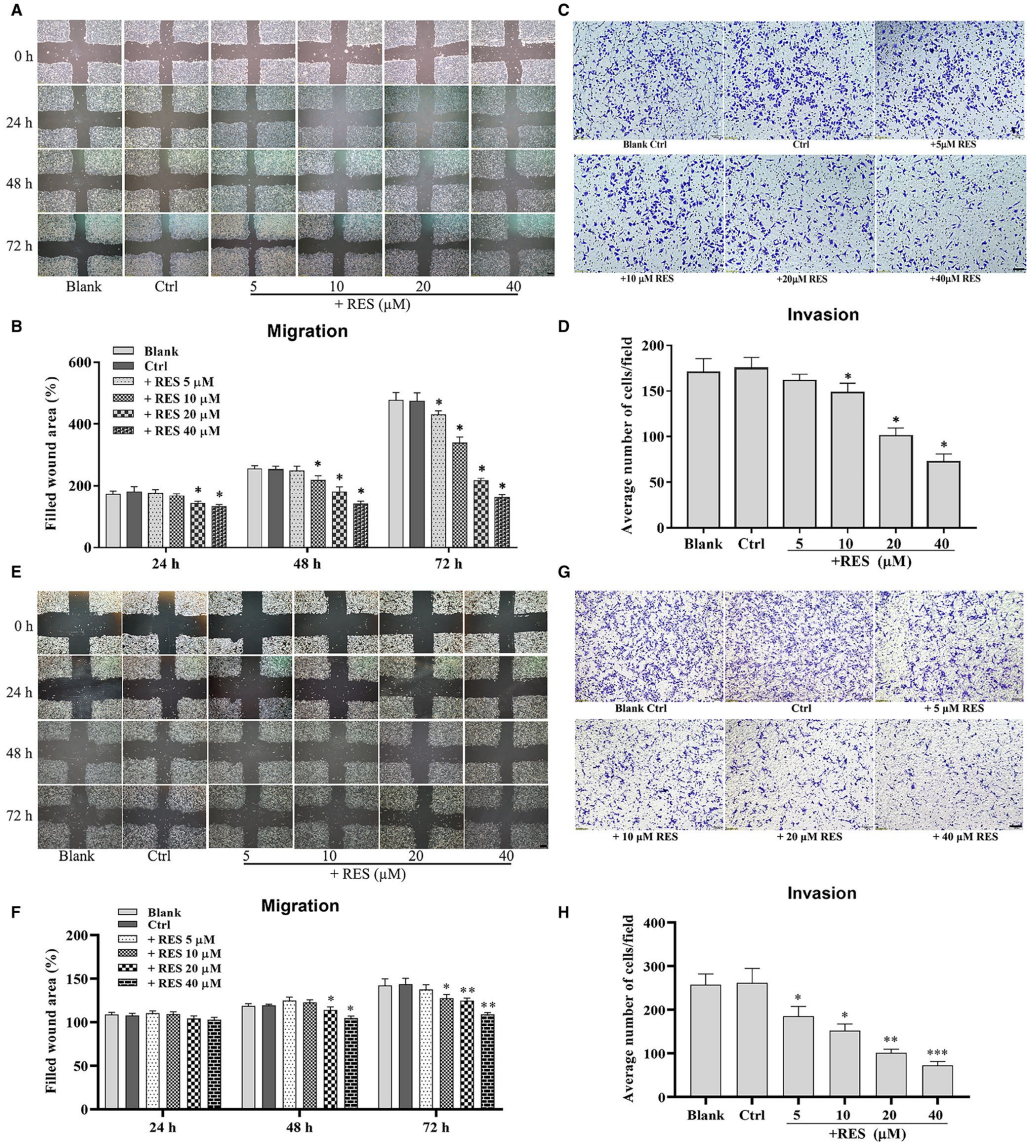
RES inhibits the migration and invasion capabilities of cervical cancer cells. The effects of RES on the migration potential of HeLa cells (A) and SiHa cells (E) were examined using a wound healing assay. HeLa cells were seeded, scratched, and treated with RES at the indicated concentrations for 0, 24, 48, and 72 h. The cells in the dishes were examined, and scratches were measured using a light microscope, scale bar =200 μm. Quantitative analysis of the scratch sizes in the wound healing assay of HeLa cells (B) and SiHa cells (F). The effects of RES on the invasion potential of HeLa cells(C) and SiHa cells (G) were examined using a Transwell assay. HeLa cells were seeded in the Transwell Boyden chambers, and then, treated with RES at indicated concentrations for 24 h. The cells that passed the Transwell chamber were stained with crystal violet and examined using a light microscope, scale bar =100 μm. (D) Quantitative analysis of the number of migrated cells in the Transwell assay of HeLa cells (B) and SiHa cells (H). *, compared with the control, *p* < 0.05; **, compared with the control, *p* < 0.01; ***, compared with the control, *p* < 0.0001

HeLa and SiHa cells were treated with RES and wound healing and Transwell assays were used to determine their migration and invasion capabilities, as well as the effects of RES on the cancer cells. The wound healing assay revealed that RES treatment resulted in a decrease in the width of scratches in HeLa and SiHa cell layers in a dose‐dependent manner (Figure 2A,B,E,F). The Transwell assay demonstrated that RES decreased the number of HeLa and SiHa cells on the lower surface of the filters in the upper chambers of Transwell (Figure 2C,D,G,H). This data suggested that RES inhibited the migration and invasion of cervical cancer cells.

**Figure 3:**
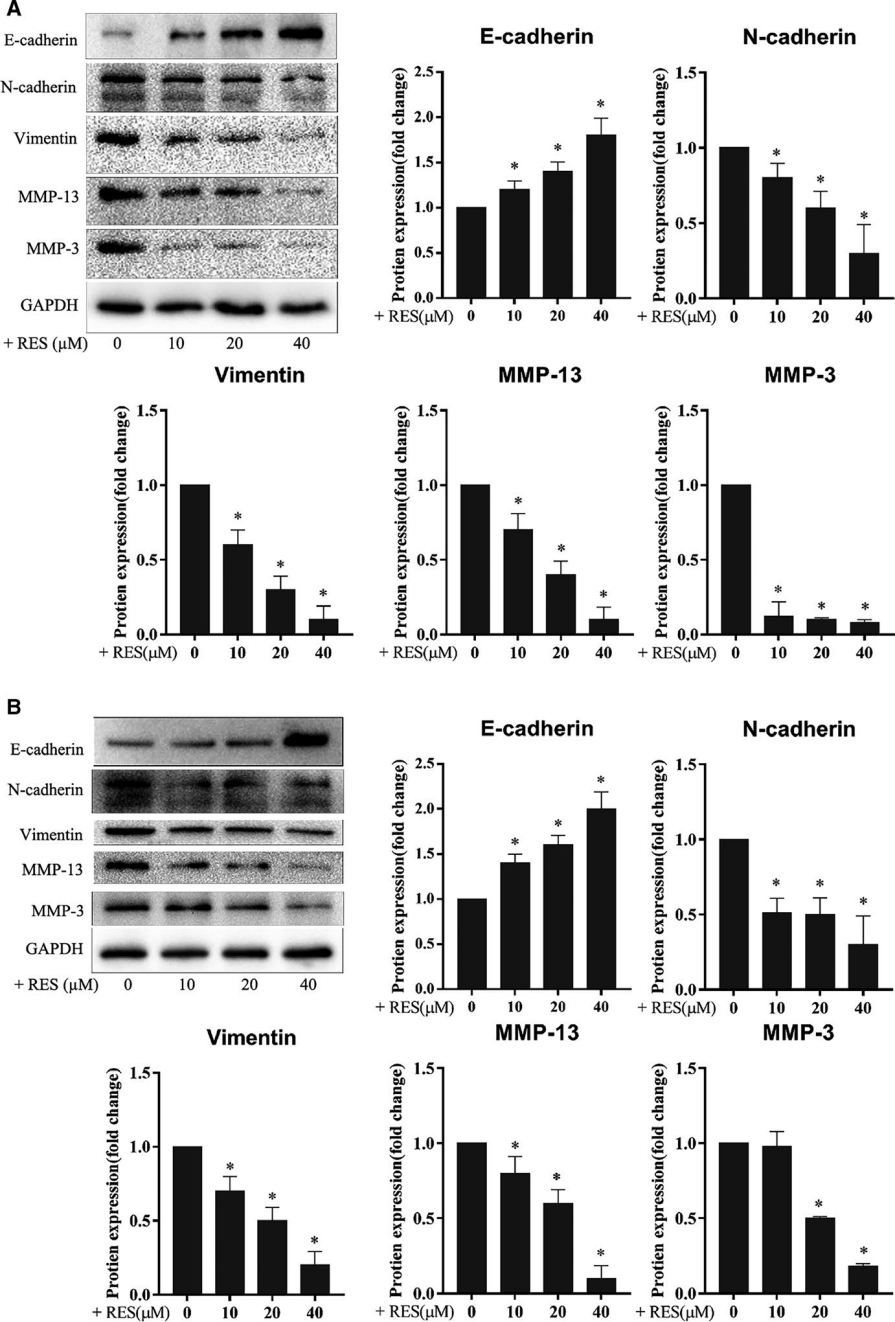
RES inhibits the expression of the EMT molecular markers and ECM degradation enzymes of cervical cancer cells. HeLa (A) and SiHa cells (B) were seeded in six‐well plates at 1 × 10^6^ cells/well, cultured for 24 h, and treated with RES at the indicated concentrations in fresh media for 24 h. The protein levels were determined using Western blot. Samples derived from the same experiment and blots were processed in parallel. *, compared with the control, *p* < 0.05

HeLa and SiHa cells were treated with RES, and the expression levels of epithelial‐mesenchymal transition (EMT) molecular markers, such as N‐cadherin, E‐cadherin, and vimentin, and extracellular matrix (ECM) degradation enzymes, such as MMP‐3 and MMP‐13, which indicated the invasion potential of cervical cancer cells were determined. Effects of RES on the EMT and ECM degradation enzymes of cervical cancer cells were also examined. Treatment with RES resulted in a decrease in the protein levels of N‐cadherin, vimentin, MMP‐3, and MMP‐13 and an increase in protein levels of E‐cadherin in HeLa cells (Figure 3A) and SiHa cells (Figure 3B), in a dose‐dependent manner. The data demonstrated that RES inhibited EMT and invasion potential of cervical cancer cells.

### 3.4 RES attenuates STAT3 phosphorylation and potentially interacts with stat3 in cervical cancer cells

**Figure 4:**
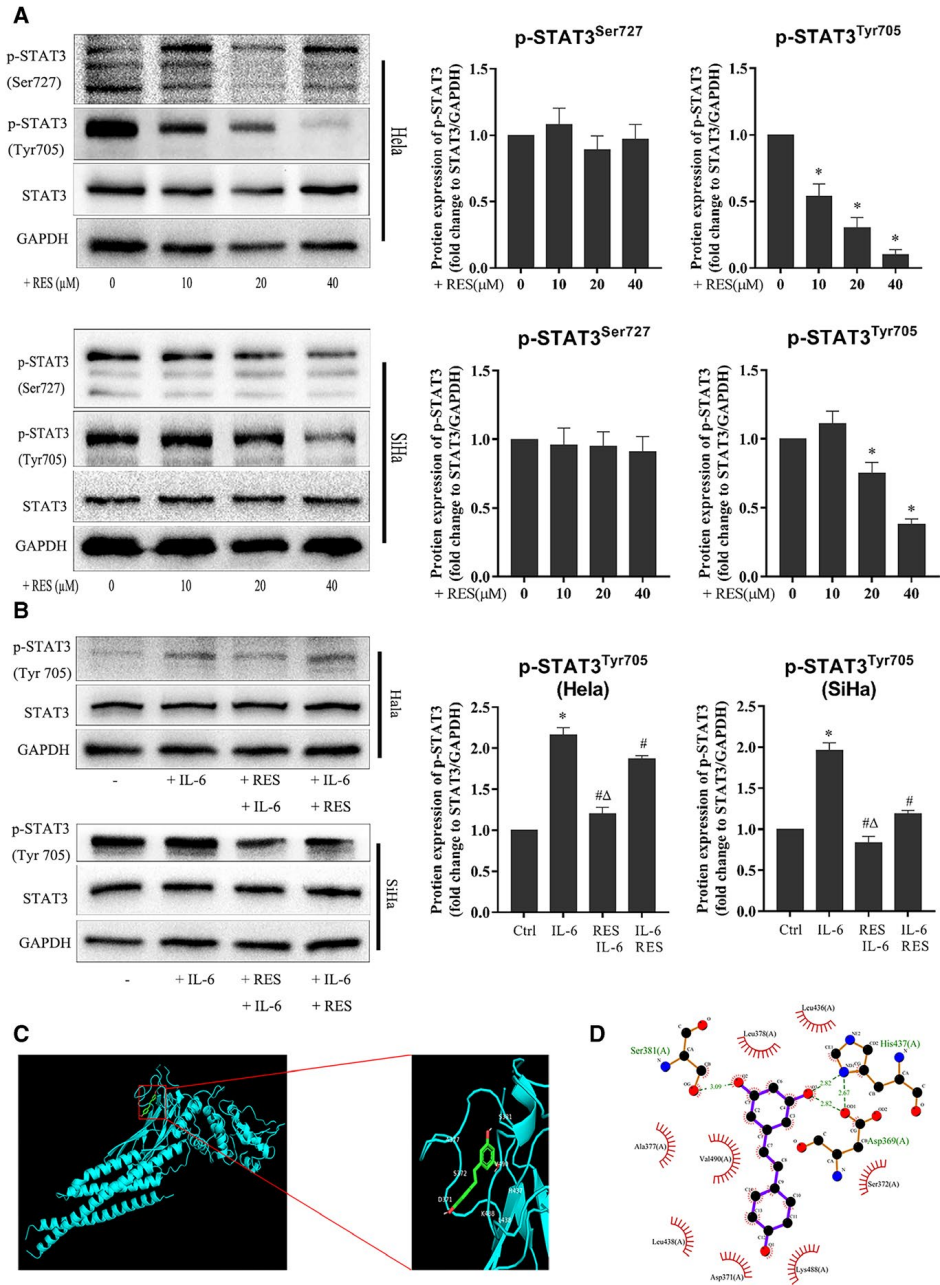
RES interacts with STAT3 and inhibits STAT3 phosphorylation in cervical cancer cells. (A) RES inhibits STAT3 phosphorylation in cervical cancer cells. HeLa and SiHa cells were seeded in six‐well plates at 1 × 10^6^ cells/well, cultured for 24 h, and treated with RES at the indicated concentrations, in fresh media for 24 h. The protein levels were determined using Western blot. Samples derived from the same experiment and blots were processed in parallel. *Compared to the 0 group, *p* < 0.05. (B) HeLa cells and SiHa cells were seeded in six‐well plates at 1 × 10^6^ cells/well, cultured for 24 h and divided into four groups. In the control group, cells were treated with a vehicle. In the IL‐6 treatment group, cells were treated with IL‐6. In the RES+IL‐6 group, cells were treated with RES for 24 h, and IL‐6 was added and incubated for another 24 h. In the IL‐6+ RES group, cells were treated with IL‐6 for 3 h, and RES was added and incubated for another 24 h. Cells were collected after treatment, and their protein levels were determined through Western blot. The samples derived from the same experiment and the blots were processed in parallel. RES 40 mM; IL‐6, 50 μg/ml. *, compared with the control, *p* < 0.05; #, compared with the IL‐6 group, *p* < 0.05; and^Δ^, compared with the IL‐6+RES group, *p* < 0.05. (C) Molecular docking between RES and STAT3. A docking model was generated using Autodock Vina (version 1.1.2). (D) The docking pocket of STAT3 was composed of Ser381, Ala377, Val490, Leu438, Asp371, Lys488, Ser372, Asp 369, His437, Leu436, and Leu378. Ser381 and His437 formed hydrogen bonds with RES

HeLa and SiHa cells were treated with RES and their STAT3 protein. Western blot was used to examine the effects of RES on the protein levels and phosphorylation status of STAT3 in cervical cancer cells. RES decreased the phosphorylation level of STAT3 at Tyr705 but not at Ser727, whereas the STAT3 protein level showed no obvious changes in HeLa and SiHa cells (Figure 4A).

HeLa and SiHa cells were treated with RES, IL‐6, and their combination, then, their STAT3 Tyr705 was detected to further examine the role of RES in the regulation of STAT3 phosphorylation in cervical cancer cells. As expected, IL‐6 increased the phosphorylation of STAT3 Tyr705 in HeLa and SiHa cells (Figure 4B). RES pretreatment or treatment decreased the IL‐6‐activated phosphorylation of STAT3 Tyr705 in HeLa and SiHa cells (Figure 4B).

**Figure 5:**
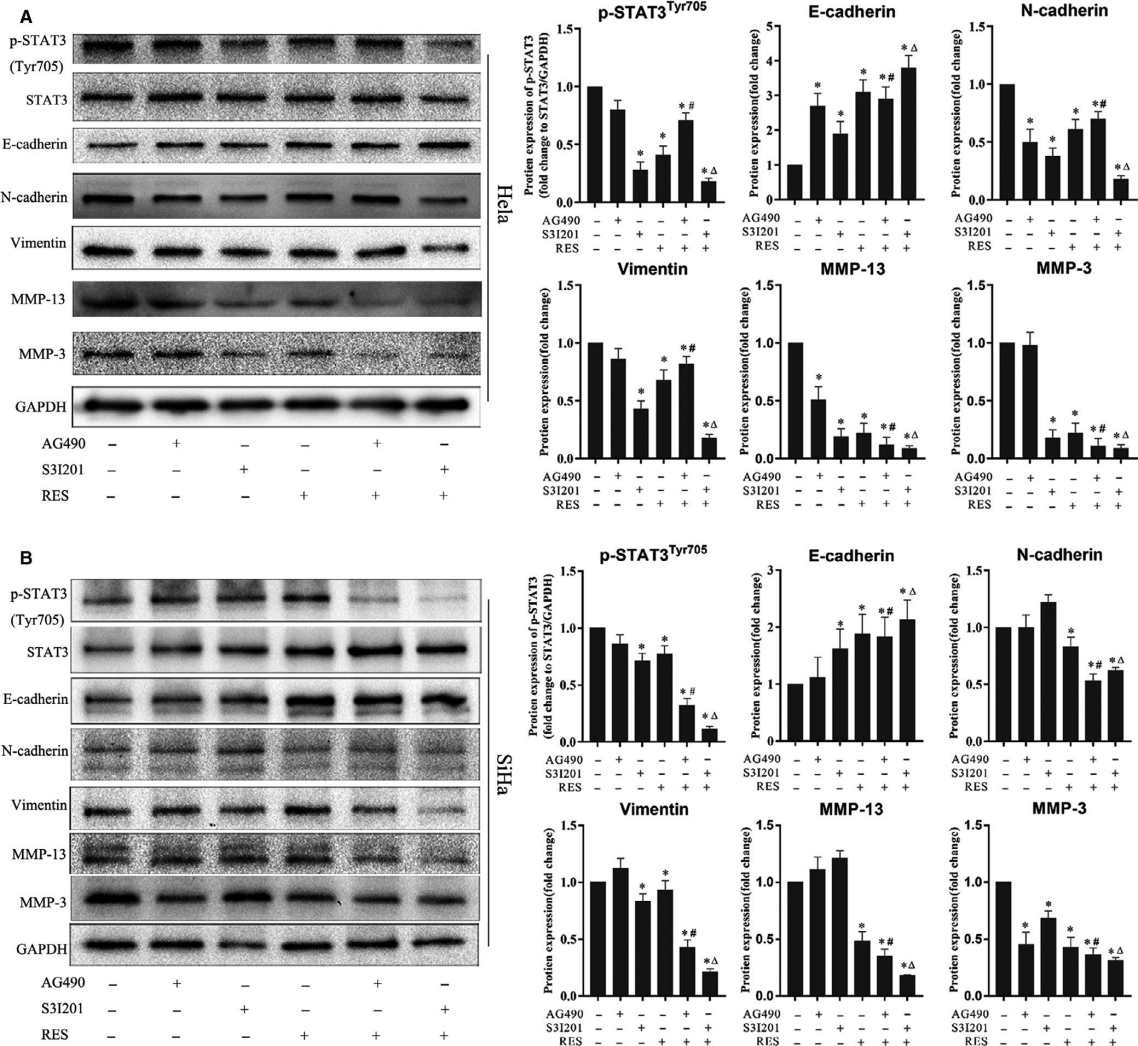
STAT3 phosphorylation reduction enhances the inhibition of the EMT molecular marker expression and ECM degradation enzymes in cervical cancer cells treated with RES. HeLa and SiHa cells were seeded in six‐well plates at 1 × 10^6^ cells/well, cultured for 24 h, and treated with S3I201 and AG490 in fresh media for 2 h. RES was then added at the indicated concentrations. After 24 h of culture, cells were collected, and the protein levels were determined through Western blot. The samples derived from the same experiment and the blots were processed in parallel. Results were quantitatively analyzed. *, compared with the control, *p* < 0.05; #, compared with AG490 treatment, *p* < 0.05; and Δ, compared with S3I201 treatment, *p* < 0.05

A structure‐based molecular docking study was performed to illustrate the potential interaction between RES and STAT3. The strongest binding sites are shown in Figure 5A with a binding affinity of −7.1 kcal/mol. The pocket was composed of Ser381, Ala377, Val490, Leu438, Asp371, Lys488, Ser372, Asp369, His437, Leu436, and Leu378. Ser381 and His437 formed hydrogen bonds with RES (Figure 4C,D).

This data demonstrated that RES inhibited the phosphorylation of STAT3 and potentially interacted with STAT3 in cervical cancer cells.

### 3.5 Reduced STAT3 phosphorylation enhances inhibitory effects of res on cervical cancer cell invasion

HeLa and SiHa cells were treated with RES and S3I201 or AG490, and the expression levels of N‐cadherin, E‐cadherin, vimentin, MMP‐3, and MMP‐13 were examined to confirm that the role of STAT3 phosphorylation was inhibited by RES on the invasion potential of cervical cancer cells. S3I201 inhibits STAT3 dimerization, DNA binding, and transcriptional activity.^40^ In addition, S3I201 inhibits the phosphorylation of STAT3 at Ser727^41^ and Tyr705.^42^ AG490 is a JAK‐specific inhibitor that can suppress STAT3 signaling by inhibiting the Tyr705 phosphorylation of the STAT3 protein.^43^ The results showed that RES, S3I201, or AG490 inhibited the phosphorylation of STAT3 ^Tyr705^ in HeLa and SiHa cells. The combined treatment of RES and S3I201 further decreased the phosphorylation of STAT3 ^Tyr705^ in HeLa cells (Figure 5A) and SiHa cells (Figure 5B). Moreover, RES, S3I201, or AG490 reduced the protein levels of N‐cadherin, vimentin, MMP‐3, and MMP‐13 and increased the protein level of E‐cadherin in HeLa and SiHa cells. Similarly, the combined treatment decreased the protein levels of N‐cadherin, vimentin, MMP‐3, and MMP‐13 but increased protein levels of E‐cadherin in HeLa cells (Figure 5A) and SiHa cells (Figure 5B). The results suggest that reduced phosphorylation level of STAT3 enhanced the inhibitory effects of RES on the invasion potential of cervical cancer cells.

**Figure 6:**
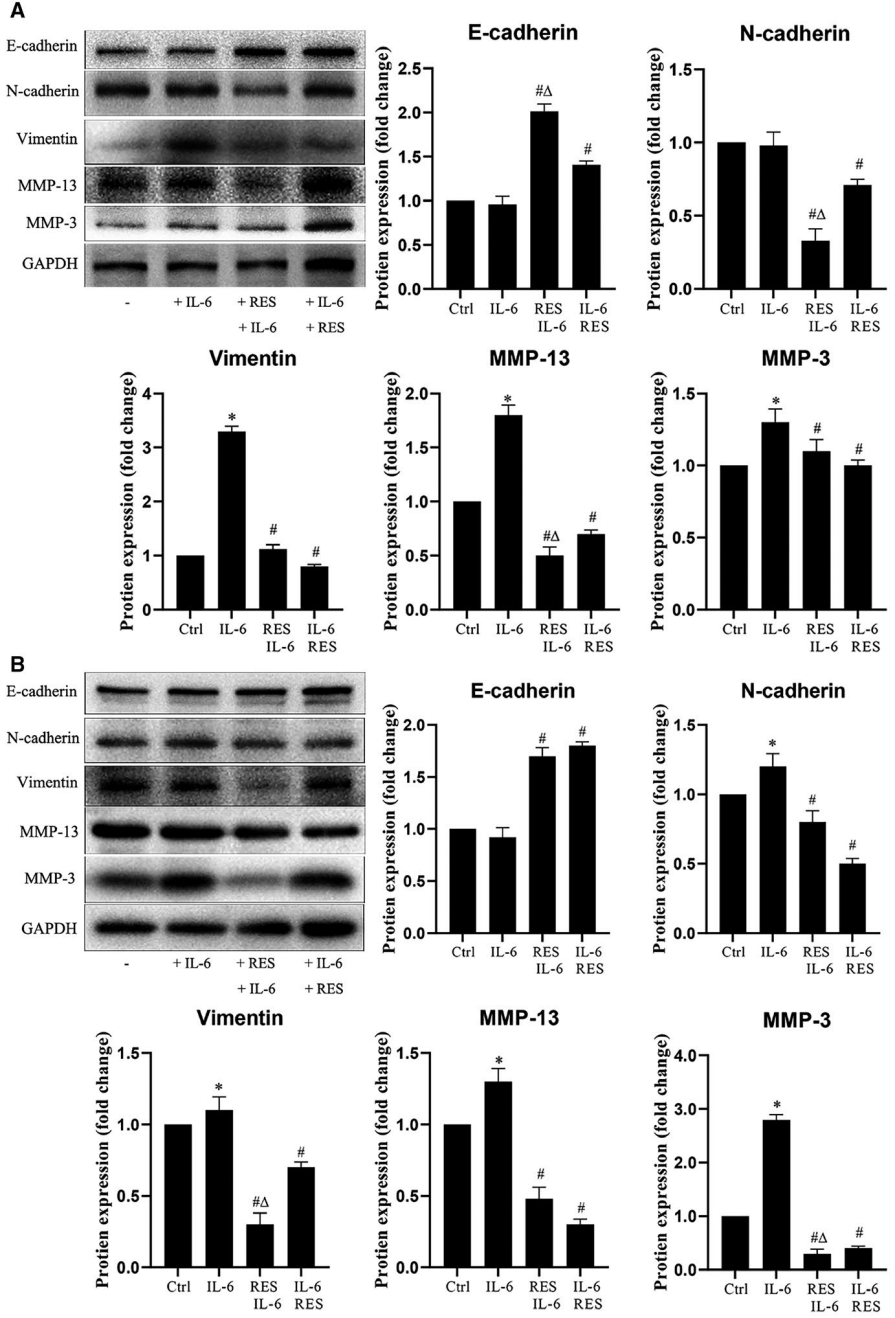
RES inhibits EMT and ECM caused by STAT3 activation. (A) HeLa cells and (B) SiHa cells were seeded in 6‐well plates at 1 × 10^6^ cells/well, cultured for 24 h and divided into four groups. Cells were treated with a vehicle in the control group. Cells were treated with IL‐6 in the IL‐6 treatment group; cells were treated with RES for 24 h in the resveratrol+IL‐6 group, and IL‐6 was added and incubated for another 24 h. Cells were treated with IL‐6 for 3 h in the IL‐6+ resveratrol group, and resveratrol was added and incubated for another 24 h. Cells were collected after treatment, and E‐cadherin, N‐cadherin, vimentin, MMP‐13, and MMP‐3 protein levels were determined through Western blot. The samples derived from the same experiment and the blots were processed in parallel. RES 40 mM; IL‐6, 50 μg/ml. Results were quantitatively analyzed. *, compared with the control, *p* < 0.05; #, compared with the IL‐6 group, *p* < 0.05; and^Δ^, compared with the IL‐6+RES group, *p* < 0.05

IL‐6 was used to activate STAT3 signaling pathway in HeLa and SiHa cells, and the expression of N‐cadherin, E‐cadherin, vimentin, MMP‐3, and MMP‐13 were examined to confirm the activation of the STAT3 signaling pathway to promote the invasion potential of cervical cancer cells. The upregulation induced by IL‐6 was prominently inhibited by RES in HeLa cells (Figure 6A) and SiHa cells (Figure 6B).

### 3.6 RES inhibits STAT3 phosphorylation, EMT, and cervical cancer cell invasion in mice

**Figure 7:**
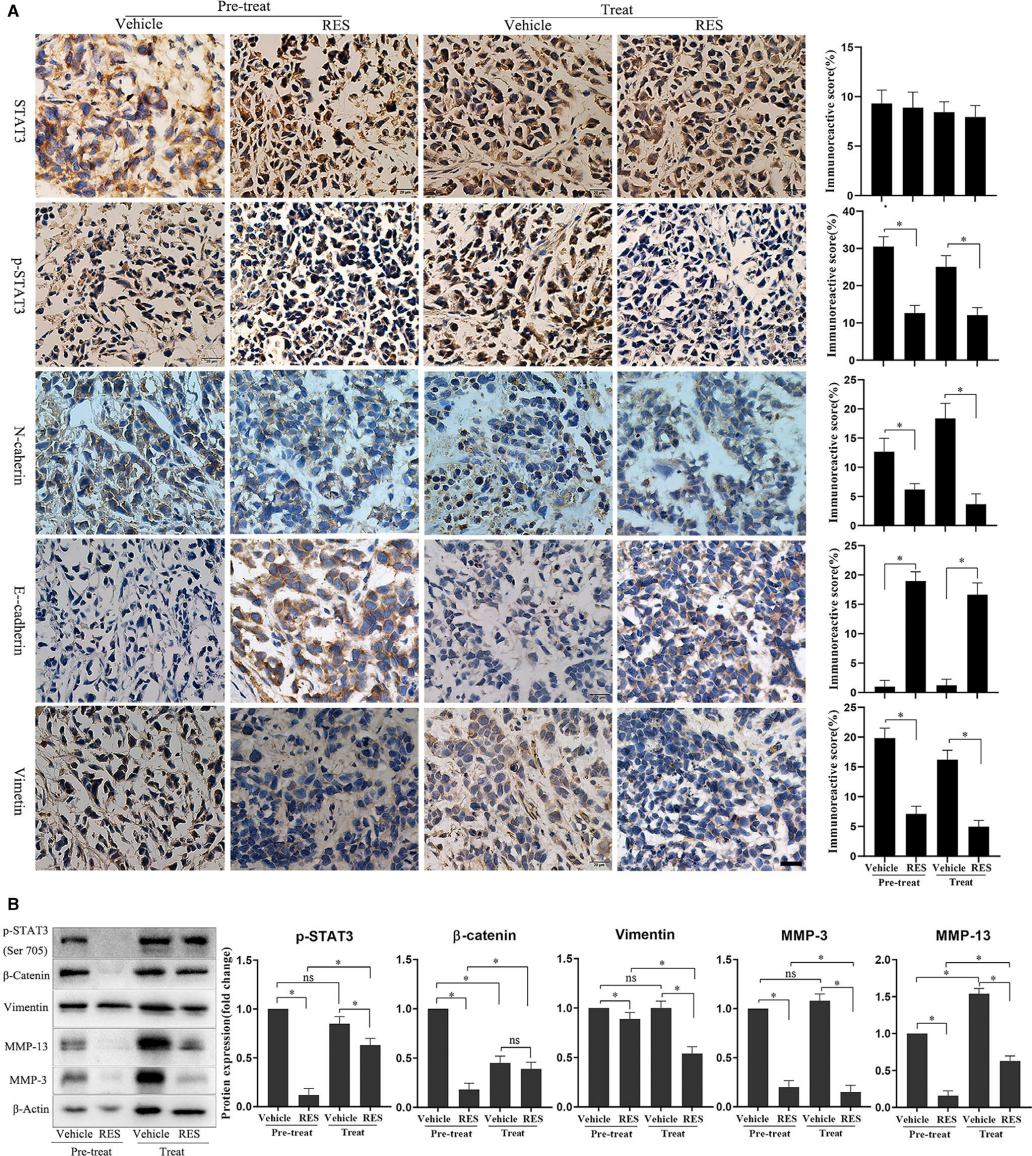
Pretreatment or treatment with RES inhibited the expression of EMT molecular markers and ECM degradation enzymes and the phosphorylation of STAT3 in cervical cancer tissues in the mouse model. (A) STAT3, p‐STAT3 (Tyr705), N‐cadherin, E‐cadherin, and vimentin in the tumor tissues grown from HeLa cells were determined through immunohistochemistry. Scale bar =20 μm. The results were quantitatively analyzed. *, *p* < 0.05. (B) p‐STAT3 (Tyr705), vimentin, MMP‐13, and MMP‐3 protein levels in the tumor tissues grown from HeLa cells were determined through Western blot. The samples derived from the same experiment and the blots were processed in parallel. The results were quantitatively analyzed. *, *p* < 0.05

The protein levels of p‐STAT3 (Tyr705), N‐cadherin, E‐cadherin, vimentin, MMP‐13, and MMP‐3 in the tumor tissues grown from HeLa cells were determined through IHC and Western blot to examine the effects of RES on STAT3 phosphorylation, EMT, and cervical cancer cell invasion. IHC results showed that the protein level of E‐cadherin increased, whereas protein levels of p‐STAT3 (Tyr705), N‐cadherin, and vimentin decreased in the tumor tissues grown from HeLa cells in the RES pretreatment and treatment groups, compared to those in the respective control groups (Figure 7A). Western blot results revealed that the levels of p‐STAT3 (Tyr705), vimentin, MMP‐13, and MMP‐3 decreased in the tumor tissues grown from HeLa cells in the RES pretreatment and treatment groups, compared with those in the respective control groups (Figure 7B). The magnitude of changes was higher in the RES pretreatment group than in the RES treatment group. These results suggest that RES inhibited STAT3 phosphorylation, EMT, and cervical cancer cell invasion in the mouse model.

## 4 DISCUSSION

Our study showed that RES inhibited the proliferation, migration, and invasion of HeLa and SiHa cells. This result was consistent with previous findings.^9^, ^10^, ^11^, ^12^, ^14^, ^15^ We first clarified that RES also inhibited cervical tumor growth of the HeLa cell xenograft in the mouse model (Figure 1A‐D). Furthermore, RES pretreatment also had inhibitory effects on the cervical tumor growth of HeLa cell xenograft in the mouse model. Its magnitude was higher than that in the RES treatment group. This finding suggests that RES exerted suppressive effects on cervical cancer that were independent of the treatment regimen. Pretreatment yielded a better outcome than the Posttreatment. Many reports have demonstrated that RES could be used as a cancer chemopreventive agent owing to its ability to induce growth inhibition, cell cycle arrest, and apoptosis in several human cancer cell lines.^44^, ^45^ Our results provide new experimental evidence for the use of RES as a cancer preventive agent.

Metastasis is a complicated biological process that involves primary tumor angiogenesis, cancer cell invasion, vascular intravasation, distant target organ extravasation, and colonization of a foreign microenvironment by invading cells. Primary epithelial cancer cells can acquire migration and invasion abilities^46^, ^47^, ^48^ with EMT. Matrix metalloproteinases (MMPs) degrade ECM around invasive cancer cells and facilitate vascular intravasation of cancer cells. E‐cadherin and N‐cadherin are the main biomarkers of EMT.^49^, ^50^ In the course of EMT, the expression levels of E‐cadherin decreases, whereas the expression level of N‐cadherin increases.^51^, ^52^, ^53^ High β‐catenin levels promote the expression of genes that facilitate EMT.^54^ High vimentin is a biomarker in the mesenchymal state of cancer cells, mediating cytoskeletal organization, and focal adhesion maturation.^55^, ^56^ MMP‐3 and MMP‐13 are proteolytic enzymes involved in the degradation of ECM around invasive cancer cells.^57^, ^58^, ^59^ In the current study, RES decreased the protein levels of N‐cadherin, vimentin, MMP‐3, and MMP‐13, and increased the protein level of E‐cadherin in HeLa and SiHa cells in a dose‐dependent manner (Figure 3A,B). The protein levels of vimentin, MMP‐13, and MMP‐3 markedly decreased in the tumor tissues grown from HeLa cells in the RES pretreatment and treatment mouse models compared to those in their respective control groups (Figure 6A,B). These results suggest that RES inhibited the metastatic potential of cervical cancer by inhibiting EMT and ECM enzyme expression.

Inhibition of STAT3 signaling plays a critical role in RES‐induced suppression of several cancer types. RES inhibits STAT3 ^Tyr705^ phosphorylation in ovarian cancer,^20^, ^21^ pancreatic cancer,^22^ head and neck tumor,^23^ osteosarcoma,^24^ colorectal cancer,^25^ colon cancer,^22^, ^23^, ^24^, ^25^, ^26^ and STAT3^S727^ phosphorylation in head and neck tumor and colorectal cancer.^23^, ^25^ Similarly, STAT3 signaling is a critical target of RES to induce apoptosis of SiHa and HeLa cells.^27^ In this study, RES inhibited phosphorylation of STAT3 at Tyr705 but not at Ser727 (Figure 4A). The STAT3 phosphorylation inhibitors AG490^43^ and S3I201^42^ enhance the effects of RES in SiHa and HeLa cells (Figure 5). The STAT3 phosphorylation activator IL‐6 antagonizes the effects of RES in SiHa and HeLa cells. RES pretreatment or treatment decreases the levels of STAT3 ^Tyr705^ phosphorylation stimulated by IL‐6 in HeLa and SiHa cells compared to those on IL‐6 treatment. However, RES pretreatment resulted in a significant reduction in IL‐6‐induced STAT3 ^Tyr705^ phosphorylation in HeLa and SiHa cells compared with that in the RES treatment group (Figure 4B). Strikingly, RES can downregulate the expression of IL‐6‐induced P‐Stat3 and its related EMT and ECM‐related biomarkers, and upregulate the expression of epithelial marker E‐cadherin (Figure 6A,B), to inhibit the metastatic potential of cervical cancer cells and play an antitumor role. In the mouse model, RES inhibited the expression of other EMT and ECM‐related biomarkers and STST3 ^Tyr705^ compared to those in the treatment group (Figure 7A,B). Furthermore, our structure‐based molecular docking study revealed that RES directly interacted with STAT3. Therefore, RES inhibited the phosphorylation of STAT3 at Tyr705 but not at Ser727 in SiHa and HeLa cells. This probably occurred through direct interaction between RES and STAT3 (Figure 4C,D).

**Figure 8:**
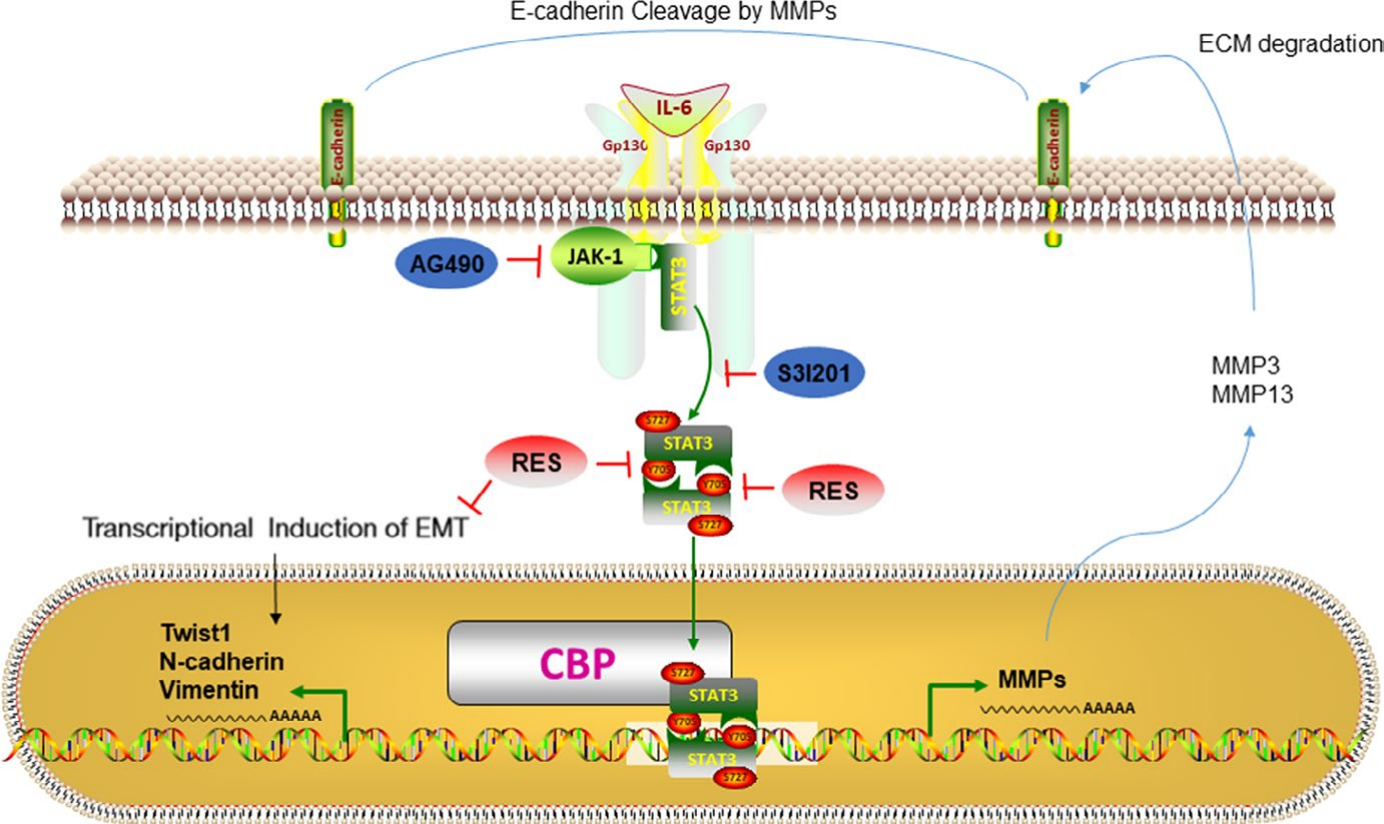
Schematic diagram of the mechanisms contributing to RES inhibiting the metastatic potential of cervical cancer cells. RES suppressed the metastatic potential of cervical cancer cells by inactivating STAT3 pathway and this effect was intensified by inhibiting of the STAT3 pathway

Studies have demonstrated that the activation of STAT3 signaling promotes metastasis of cervical cancer cells.^60^, ^61^, ^62^ S3I201 inhibits STAT3, while AG490 is an inhibitor of the JAK upstream of STAT3. Our study indicated that RES had similar effects to S3I201 and AG490, resveratrol combined with S3I201 or AG490, and consistently decreased phosphorylation levels of STAT3 ^Tyr705^ in HeLa and SiHa cells. RES, S3I201, and AG490 treatment resulted in a decrease in protein levels of N‐cadherin, vimentin, MMP‐3, and MMP‐13. There was also an increase in the protein level of E‐cadherin in HeLa and SiHa cells. The combined treatment of RES and S3I201 further decreased the protein levels of N‐cadherin, vimentin, MMP‐3, and MMP‐13 and increased the protein levels of E‐cadherin in HeLa and SiHa cells (Figure 5A,B). This also indicates that RES mainly targets STAT3 and inhibits its phosphorylation. The combined treatment of RES and S3I201 further decreased the phosphorylation level of STAT3 ^Tyr705^ in HeLa and SiHa cells. These findings corresponded to those of EMT and ECM enzyme biomarkers. A similar association was observed in STAT3 ^Tyr705^ phosphorylation, and EMT and ECM enzyme biomarkers in tumor tissues grown from HeLa cells in the mouse model (Figure 7A,B). Our findings suggest that STAT3 is intrinsically targeted with the cell's response to RES. This effect was intensified by inhibitor STAT3 pathway, as shown in Figure 8.

In conclusion, the current study indicates that RES inhibits growth of cervical cancer, and EMT and ECM degradation by inhibiting STAT3 ^Tyr705^ phosphorylation. Therefore, RES is a potential chemotherapeutic and a natural chemopreventive compound for treating cervical cancer.

## CONFLICT OF INTEREST

The authors declare no competing financial interests.

## AUTHOR CONTRIBUTION

SXD was responsible for designing the study, data collection and analysis, and preparing the graphs, and was a major contributor to writing the manuscript; XQQ, ZL and XLX were responsible for performing the experiments; ZQ was responsible for the statistical analysis; XHX processed the charts and tables in the revision process of the later articles; WXB and JN contributed to the critical review of the manuscript; SM and FP supervised and contributed to the critical review of the manuscript. All authors have read and approved the manuscript.

## ETHICAL APPROVAL

Mouse xenograft experiments in this study were complied with the ARRIVE guidelines and were conducted in accordance with the U.K. Animals (Scientific Procedures) Act, 1986 and associated guidelines. This study was approved by the Ethical Committee for Animal Experimentation of Xiangyang No. 1 People's Hospital (NO. 2017DW006).

## Supporting information

Fig S1

Supplementary Material

## Data Availability

Data that support study findings are available with the corresponding author upon reasonable request.
