# Supplementary material for: Resveratrol suppresses the growth and metastatic potential of cervical cancer by inhibiting STAT3Tyr705 phosphorylation
Source: Cancer Med. 2020 Oct 10;9(22):8685–700. doi: 10.1002/cam4.3510 (PMC7666735; doi:10.1002/cam4.3510)
Supplement: Supplementary file 2 — Supplementary Material [file CAM4-9-8685-s002.docx]

**Suppl. Figure 1 RES inhibited the proliferation of cervical cancer cells.** (A) HeLa cells were treated with RES at indicated concentrations for 24, 48, and 72 h. Cell proliferation was determined using a CCK-8 kit. (B) IC_50_ of RES that inhibited HeLa cell growth. (C) Colony formation of HeLa cells treated with RES. HeLa cells were treated with RES at indicated concentrations for 14 days. (D) Quantitative analysis of the colony formation of HeLa cells treated with RES. *, compared with the control, *P*<0.05.
